# Long-Lasting Effects of Undernutrition

**DOI:** 10.3390/ijerph8061817

**Published:** 2011-05-26

**Authors:** Vinicius J. B. Martins, Telma M. M. Toledo Florêncio, Luciane P. Grillo, Maria do Carmo P. Franco, Paula A. Martins, Ana Paula G. Clemente, Carla D. L. Santos, Maria de Fatima A. Vieira, Ana Lydia Sawaya

**Affiliations:** 1 Department of Physiology, Federal University of São Paulo, Rua Botucatu, 862, Edifício de Ciências Biomédicas, 2°andar CEP 04023-060 São Paulo, SP, Brazil; E-Mails: mdcfranco@nefro.epm.br (M.C.P.F.); anapaula_grotticlemente@yahoo.com.br (A.P.G.C.); carla-dls@hotmail.com (C.D.L.S); alsawaya@unifesp.br (A.L.S.); 2 Department of Nutrition, Federal University of Alagoas, Rua Hélio Pradines, 225/301 Ponta Verde, CEP 57035-220 Maceió, Alagoas, Brazil; E-Mail: telmatf_al@hotmail.com; 3 Professional Masters Program in Health and Work Management, Vale of Itajaí University, Rua Uruguai 458, Bloco 25 B, Sala 402, Centro, CEP 88302-202 Itajaí, SC, Brazil; E-Mail: grillo@univali.br; 4 Department of Health Sciences, Federal University of São Paulo, Av. Ana Costa, 95 Vila Matias CEP 11060-001, Santos, São Paulo, Brazil; E-Mail: paula.martins@unifesp.br; 5 Nutrition College, Federal University of Pelotas, Campus Universitário, CP 354, Pelotas, RS, Brazil; E-Mail: fvieira.nut@gmail.com

**Keywords:** undernutrition, hypertension, body composition, dyslipidaemia, insulin, energy expenditure

## Abstract

Undernutrition is one of the most important public health problems, affecting more than 900 million individuals around the World. It is responsible for the highest mortality rate in children and has long-lasting physiologic effects, including an increased susceptibility to fat accumulation mostly in the central region of the body, lower fat oxidation, lower resting and postprandial energy expenditure, insulin resistance in adulthood, hypertension, dyslipidaemia and a reduced capacity for manual work, among other impairments. Marked changes in the function of the autonomic nervous system have been described in undernourished experimental animals. Some of these effects seem to be epigenetic, passing on to the next generation. Undernutrition in children has been linked to poor mental development and school achievement as well as behavioural abnormalities. However, there is still a debate in the literature regarding whether some of these effects are permanent or reversible. Stunted children who had experienced catch-up growth had verbal vocabulary and quantitative test scores that did not differ from children who were not stunted. Children treated before 6 years of age in day-hospitals and who recovered in weight and height have normal body compositions, bone mineral densities and insulin production and sensitivity.

## 1. Introduction

Undernutrition is defined by an insufficient provision of energy and nutrients, such as good quality protein with an adequate balance of essential amino acids, vitamins and minerals, and an inability to meet the requirements of the body to ensure growth, maintenance, and specific functions [1]. It should be noted that in this review, the term undernutrition is used in its broadest sense, that is, to mean inadequate nutrition, which includes an insufficient quantity of food as well as an inadequate consumption of food in qualitative terms. This is important because the consumption of energy-dense foods with poor nutrient quality is a problem today that also affects developing countries and the poorest populations. This new nutritional situation further increases the impact of the long-lasting effects of undernutrition.

The prevalence of undernutrition exceeds 900 million people across the World [2]. Child and maternal undernutrition is responsible for approximately 3.5 million deaths in children below the age of 5 years and for 35% of the disease burden in this age group [3]. Stunting, low weight and low birth weight (LBW) are together responsible for 2.2 million deaths among children (<5 y) worldwide and for 21% of disability-adjusted life years (DALYs) [3]. One DALY is equivalent to the loss of one year of healthy life.

Apart from the serious consequences on a person's health, the economy is also affected by undernutrition, because the high prevalence of this condition hinders economic development and perpetuates poverty both directly, through a loss of productivity due to poor physical condition, and indirectly, through poor cognitive function and learning deficits. Furthermore, undernutrition increases health expenses [4]. On the other hand, the worldwide prevalence of obesity has been increasing in countries with a low per capita income, a state that coexists with undernutrition [5]. This article examines undernutrition in the early years of life and its long-term consequences, such as the risk of developing obesity, diabetes, hypertension and, consequently, cardiopathies, which are already evident among adolescents as well as adult residents in the slums of Brazil. We also describe some of the studies carried out thus far illustrating the positive effects of nutritional recovery on weight and height, body composition, and the metabolism of glucose and insulin.

## 2. Nutritional Transition

The concept of nutritional transition was developed in an attempt to describe the changes in the worldwide prevalence of undernutrition and obesity according to the changes seen over the last few decades in lifestyles and standards of living [6,7]. Under this concept, the initial stages of nutritional transition are characterised by serious food insecurity and poverty and an increased prevalence of people who are underweight, wasting and stunting, as well as a very low prevalence or the absence of obesity. Improvements in the socio-economic conditions associated with growing urbanisation favour excess weight gain in some segments of the population, although undernutrition persists, above all, in rural areas. This contrast between a high and low prevalence of obesity in urban and rural areas, respectively, has been well documented in Latin America [8,9]. Finally, at a more advanced stage of nutritional transition, the prevalence of undernutrition is very low, while the prevalence of obesity and chronic diseases is raised. However, a large number of variables affect the nutritional transition, modulating the effects of urbanisation on dietetic standards and decreases in physical activity. Among these variables, one that cannot be ignored is the fact that urbanisation has been accompanied by a very high increase in the number of slum dwellers. The growth of the urban population, particularly in emerging countries, has been associated with the growth of slums at a faster pace than the rest of urban population. Large groups of the population experience unhealthy living conditions with a high frequency of infections and inadequate nutrition. All of these factors, among others, modulate the way in which the organism controls the flow of body energy, and consequently, its response to environmental insults.

## 3. Coexistence of Undernutrition and Obesity

Overweight and obesity have reached epidemic proportions, affecting around 1.5 billion adults and 200 million children of school age throughout the World, making this the first generation with a predicted life expectancy lower than that of their parents [10,11]. In the past, obesity was found only in affluent populations with an abundant energy intake, whereas obesity is now frequently associated with stunting in countries with a low per capita income and high food insecurity [12]. The co-existence of low body weight and/or stunting and obesity in poor populations has been described in Asia, China, Africa and, above all, in Latin America [13–15].

One of the first studies on poor urban populations was carried out by our research group in the late 1990s using a census designed to investigate the socio-economic profile and nutritional status of 2,411 slum-dwellers in São Paulo, Brazil [16]. We found a prevalence of 30% stunting or low weight in children (0–10 y) and 15% obesity in adults (>18 y). Nine percent of homes presented at least one resident with low weight and/or stunting and one obese individual.

The same profile has now been observed among poor children and adolescents in other countries. For example, a South African study [17] randomly selected children and adolescents (1–20 y) from a rural community with high levels of poverty to investigate the prevalence of stunting, overweight/obesity and the risk of metabolic diseases. Stunting was highly prevalent (18%) in children (1–4 y). The prevalence of overweight and obesity was moderate in early childhood and low at the end of childhood. The prevalence of overweight and obesity increased progressively in girls from the age of 10 years, reaching 10% and 15%, respectively, in girls between the ages of 10 and 16, and affecting 15% and 25%, respectively, of girls between 17 and 20 y. When stratified for pubertal stage, girls at stage five exhibited a prevalence of overweight and obesity of 35%, while among boys, the prevalence did not reach 1%. A national survey carried out in Mexico [18] found a relation of 6.2% between mothers with central adiposity and children with stunting. This phenomenon was more prevalent in rural localities and among indigenous families. Moreover, a study of poor Mexican children living in rural areas [19] found that obesity and undernutrition coexisted in the same environment with a high prevalence of stunting and overweight. All of the families lived in poverty with a large number of dwellers per home. More than 30% of the families were of indigenous origin. Functional drainage systems were present in less than 20% of the community. The prevalence of stunting in children (4–5 y) was 21.3% for the non-indigenous population and 42.7% for the indigenous population. The prevalence of overweight/obesity among stunted children was twice as high among the indigenous children as in their non-indigenous counterparts (12.1% *vs.* 5.9% respectively). The most associated factors were younger mothers of short stature, fewer years of education, worse socio-economic status, less awareness of social status and larger household size. Similarly, this type of association was observed among poor children in China [20]. Of 453 overweight children (0–5 y), 57.6% exhibited stunting (height-for-age, HAZ < −2), 41.0% showed a height for age z score between −2 and 2 and only 1.4% presented a height for age z score >2. The prevalence of stunting among all of the children was 30%, with this being the most serious and prevalent problem, while the prevalence of underweight was 10%.

One study carried out in Alagoas, a very poor region of Brazil, found a similar coexistence of undernutrition and obesity [21]. A total of 315 families were surveyed. Housing conditions were of an extremely poor standard: the majority of the families lived in plastic shacks with only one room and one household appliance, almost all of the dwellings lacked floor coverings, most dwellings had no water supply and the families used untreated water. Almost all of dwellings were without a bathroom or refrigerators. Children (<10 y) showed a high prevalence of wasting plus stunting, and most were badly affected (20%). Among adults, there was a higher prevalence of being overweight (25%) than of being underweight (20%). The prevalence of being both underweight and overweight was higher in women than in men, and in the former, the prevalence of being overweight was approximately twice that of the latter. Of the stunted individuals, 30% were overweight and 16% were underweight.

The diets of the adults [22] were surveyed to investigate the relationship between their food intake and their nutritional status (n = 532, >18 y). The mean energy intake was 63%, below the Recommended Dietary Allowances (RDA), and after adjustment for light activity and short stature, the values were around 70% and compatible with the wider panorama of undernutrition present in the population. Among the stunted group, undernourished men were shown to have a tendency of lower energy intake (5,882 kJ) in comparison to those who were obese (7,226 kJ). On the other hand, among the stunted women, energy consumption was shown to have no relation to nutritional status, as similar energy consumptions were found for undernourished and obese women, which were 4,527 kJ and 4,686 kJ, respectively. These results raise the possibility that the high prevalence of overweight/obesity, in particular among stunted women, was not associated with excessive energy consumption, but instead with an insufficient intake, if RDA values are considered to be a reference for adequate intake. On the other hand, it is important to consider that a positive energy balance must have effectively occurred to cause obesity. We can therefore suppose that the RDA values might be very high for this population due to their smaller body size, in spite of the correction made for short stature and low energy expenditure. One question to consider is the accuracy of the food consumption measurements. Dietary recall inquiry was applied to all individuals at home, on any given day, to provide information on the respondents' food intake during a single 24-hr period using a standardised manual with photographic records for dietary inquiries, which illustrated the different servings shown in grams and household measures. In a sub-sample, a 3-day inquiry was also done. As a whole, the usual diet was quite consistent (number of preparations at dinner = 21, for example), basically made up of coffee with sugar, bread and margarine, beans and rice, stewed chicken, maize floor and chicken eggs. No considerable out-of-home-foods were found, which is consistent with the level of poverty. For this reason, we believe that food intake measurements were accurate due to the lower influence of those factors known to interfere in the quality of food intake measurements, such as out-of-home uncontrolled food consumption and a diet of large variability. Another factor to be considered that could explain the presence of overweight/obese people found in this population is the amount of fat in their diets. The results did not reveal high fat intake or differences in this macronutrient for stunted and obese men and women when compared to their normal or undernourished counterparts (25% on average). The authors concluded that the obesity found in the stunted individuals could not be explained by a higher fat intake. On the other hand, it is possible that physical activity was particularly low. In addition, the unemployment rate was very high (81.6%). The most vigorous physical activities described were occasionally walking long distances to get and carry water to the household and the hand washing of clothes done by women. For this reason, other mechanisms may be responsible for the positive energy balance, such as a considerable decrease in energy expenditure for physical activity. This was found especially in studies with doubly labelled water in stunted adolescent girls [23]. Finally, it is possible that other factors may have contributed to the differences in body mass index (BMI) observed in women with short stature/low weight in comparison to those with short stature/obesity. One possibility could be an increase in energy expenditure in relation to energy intake due to a higher frequency of infections and parasites among those with low weight.

The profile described for this population was also observed in a nationally based investigation [24]. Among women (>20 y) from families that received up to 25% of the minimum salary (equivalent to $18 dollars/month), 32.1% were overweight and 8.8% were obese. A high prevalence was found among women who received between 25% and 100% of the minimum salary, with 40% of these women being overweight and 13% being obese. Men of lower income classes presented a lower prevalence of overweight and obesity in comparison to women. Furthermore, the total number of calories available per household for consumption in the population with an income of up to 25% of the minimum salary was 1,486 kcal per capita, while for the population with an income above 5 times the minimum salary, 2,075 kcal calories were available per capita [24]. More recently a similar study showed that 35.5% of families reported that the quantity of food consumed over the course of a month was either normal or occasionally insufficient [25].

One important question to point out is how obesity is evaluated in individuals with short stature, because for any given weight, the BMI will be higher in people who are shorter. For this reason, the diagnostic of obesity must be confirmed by other methods, such as bone mineral density (BMD) or waist-to-hip-ratio. This evaluation was done in a series of studies on adolescents [26] and adults [22] by us as well as other authors. These studies have confirmed that there are alterations in the body composition of stunted people with high fat accumulation. Higher co-morbities were also found in obese/short individuals and are discussed below.

## 4. Aetiology of Stunting

There are many genetic and environmental factors that modify stature. Growth hormone (GH) deficiency [27], impaired kidney function [28], psychosocial deprivation [29] as well as undernutrition can cause stunting. Although it is well known that stature has a genetic basis, there is increasing evidence of the strength that environmental factors, such as poverty and a high frequency of infections, have on the determination of final stature. Some authors have suggested that environmental conditions are stronger determinants of stature compared to genetics in adverse conditions [30,31]. Nutritional stunting due to poor living environments is estimated to affect 178 million children (<5 y) in developing countries and is the most prevalent form of nutritional deficit in the World, corresponding to 24.1% of cases [3]. Nutritional stunting is a combination of many factors, with the most important being intra-uterine and maternal undernutrition, inadequate quality or quantity of complementary foods during infancy, and an impaired absorption of nutrients caused by intestinal infections and parasites [32]. A study carried out in Brazil showed that the two main risk factors associated with stunting in the household environment were the absence of flooring in all rooms (b = 0.739, OR = 2.1, p = 0.027) and the absence of a water tap (b = 0.489, OR = 1.6, p = 0.042). The likelihood of a child or adolescent (0–18 y) presenting nutritional stunting was doubled when there was no flooring in all of the rooms and increased by 60% when there was no piped water supply in the home [33].

Numerous studies have shown that undernourished children present altered function in the GH–insulin-like growth factor (IGF) axis, with a raised concentration of GH but decreased plasma levels of IGF-1 [34,35]. This may be caused by the resistance to GH induced by undernutrition in the liver, reducing the synthesis of IGF-1, which leads to an increase in plasma GH, because IGF-1 acts in the central nervous system to control the synthesis of GH via negative feedback [36–39]. Three factors appear to control the resistance to GH in undernutrition: an elevated concentration of cortisol, a reduced concentration of insulin and a decrease in the amount of essential amino acids in the blood. The reduction in the IGF-1 concentration is the main factor responsible for the slowed growth in undernourished children. IGF-1 is also associated with the growth and differentiation of organs and has important effects on myelination in the brain because it stimulates an increase in the expression of genes associated with myelin and also causes an increase in the number of oligodendrocytes and neurons [40]. GH and IGF-1 are also important in the development and normal functioning of the immune, reproductive and cardiovascular systems [41]. Food intake and nutritional status are the main regulators of IGF-1, and due to this marked sensitivity, the assay of the blood concentration of IGF-1 can be employed as an indicator of the nutritional status in children with reduced growth [38,42] and as an indicator of dietary protein quality [36].

The long-term deleterious consequences of stunting have been mostly attributed to cortisol, in which the blood concentration is increased in undernutrition. The elevated cortisol levels seen in undernutrition are secondary, at least in part, to the fall in the rate of metabolic clearance. Stress may help to maintain the elevated concentrations, suppressing the circadian variation in cortisol secretion as a result of the persistent stimulation of the hypothalamic-pituitary-adrenocortical (HPA) axis and the consequent hypersecretion of corticotrophin releasing hormone (CRH) and adrenocorticotropin hormone (ACTH). Raised concentrations of cortisol and ACTH decrease insulin release, promote resistance to its peripheral action, favour hepatic gluconeogenesis and the production of glucose, stimulate lipolysis and inhibit the IGF-1-dependent effects of GH on growth. All of these events lead to a conservation of substrates and consequently, to stunting. Furthermore, in the short term, this hormonal profile is considered to be a survival strategy that leads to a redirecting of body energy flow, favouring more important tissues, such as the nervous system and the liver in detriment to lean mass and adipose tissue.

## 5. Energy Expenditure

Studies have shown that the total daily resting metabolic rates (RMR) of stunted children were lower than that of nonstunted children of the same age, indicating that stunting leads to a reduction in resting energy expenditure [43,44]. This reduction was due to the children's smaller sized metabolic active tissue, because no significant differences in RMR could be detected after differences in lean body mass were controlled for. Another important finding in these children was a higher respiratory quotient (RQs) in the fasting state and at 30 min after a meal [44]. These findings show that the stunted children had impaired fat oxidation compared with the nonstunted control children living in the same environment. Thus, their metabolism was somehow altered towards fat conservation. In one cross-sectional study of slum children from São Paulo (8–11 y) [23], the free-living total energy expenditure (TEE) was measured over 7 days by using the double-labelled water method. The results indicated that stunting was not associated with a low TEE, because after adjustment for body weight or fat-free mass and fat mass, there was no significant difference in the TEE between the stunted and nonstunted subjects. We did find, however, that girls had significantly lower TEEs than did boys, independent of body composition differences between sexes, a finding that may explain the greater susceptibility to obesity for poor girls than for boys described previously [23]. To further investigate this issue, we did a 36-month prospective study on the relationship between stunting, RMR and weight gain [45] in girls with stunting and girls with a normal stature (7–11 y).

**Figure 1.**
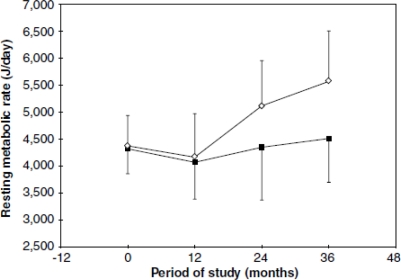
Resting metabolic rate (J/day) (mean7s.d.) over the follow-up time (nonstunted ⋄; stunted ▪). ANOVA: group factor (F (1.20) = 3.04; P = NS), time factor (F (3.60) = 12.7; P < 0.001), and interaction factor (F (3.60) = 4.7; P = 0.005). Reprinted with permission from *Eur. J. Clin. Nutr.* [45].

**Figure 2.**
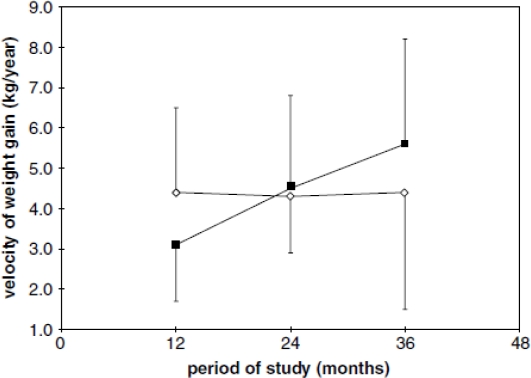
Rate of weight gain/y (mean7s.d.) over the follow-up time (nonstunted ⋄; stunted ▪). ANOVA: group factor (F (1.24) = 0.01; P = NS), time factor (F (2.48) = 3.99; P = 0.02), and interaction factor (F (2.48) = 3.99; P = 0.002). Reprinted with permission from *Eur. J. Clin. Nutr.* [45].

From 24 months of follow-up, the girls in the stunted group presented a significantly lower RMR compared to the normal stature group (Figure 1), likely as a consequence of a lower increase in lean body mass (LBM). However, other mechanisms may be acting in the conservation of energy and/or body fat, because the speed of weight gain in the stunted girls was significantly higher compared to those of normal stature (Figure 2). It appears, therefore, that in environmental conditions where the consumption of energy and nutrients is insufficient or inadequate, the organism prefers to reduce growth, energy expenditure and fat oxidation but to increase weight gain.

Many hormones are known to act by reducing the energy expenditure in people with undernutrition, and a decrease in the concentration of tri-iodothyronine represents an important energy-saving factor [46]. In acute undernutrition, there is a reduction in the total serum concentration of T3 and T4 due to decreased plasma proteins, although euthyroid status persists [47]. However, prolonged undernutrition disrupts the adaptive mechanisms, resulting in hypothyroidism with a low concentration of free T3 (the active form of the hormone) and an increase in rT3 (inactive form). The reduction in T3 reduces thermogenesis and oxygen consumption, which enables a greater conservation of energy when faced with a scarcity of the substrate. These effects appear to be regulated peripherally in the organism rather than by the central nervous system, because the response of the thyroid stimulating hormone (TSH) to stimulation by thyrotropin releasing hormone (TRH) is normal [48]. Indeed, work by our research group in obese women of short stature showed that they had significantly lower plasma concentrations of T3 compared to obese women without short stature [47].

## 6. Body Composition

**Figure 3.**
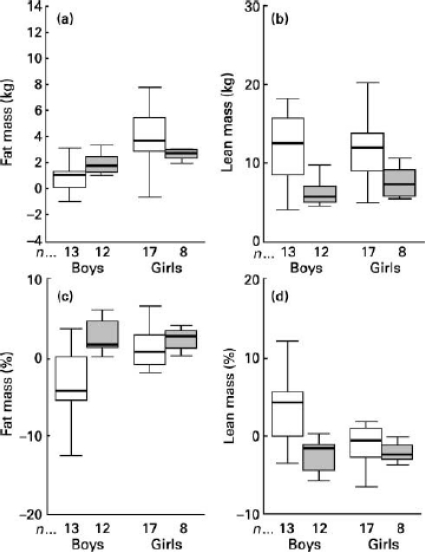
Changes in body composition of stunted ( 
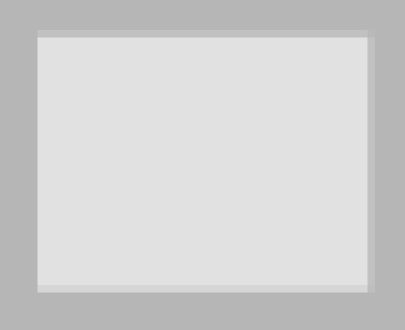
) and nonstunted children (□) (overall group) between the two study visits. (**a**) fat mass; (**b**) lean mass; (**c**) fat mass percentage; (**d**) lean mass percentage. The boxes represent the interquartile ranges, which contain 50% of values; the whiskers are the highest and lowest values (excluding outliers), and the line across each box indicates the median. Reprinted with permission from *Brit. J. Nutr.* [26].

The changes in body composition that favour the accumulation of body fat in undernourished individuals become evident during puberty. To assess the impact of stunting on the distribution of fat in adolescence, 11-year-old Senegalese girls were monitored up to the age of 15 [49]. The adolescents with stunting presented an increase in the bicipital and subscapular skinfolds, suggesting a greater deposition of subcutaneous fat in the upper part of the body. In addition, stunted girls exhibited a tendency to accumulate subcutaneous fat in the trunk when compared to the girls without stunting. Martins *et al.* [26], in a prospective 3y study, evaluated body composition assessed by dual-energy X-ray absorptiometry (DXA) in fifty boys and girls with stunting or normal stature, aged 11 to 15 y, who were living in slums in the city of São Paulo, Brazil. The stunted boys and girls accumulated more body fat and gained less lean mass (Figure 3).

Another study done by our research group showed that, compared with children without stunting, the body fat in stunted children is not distributed evenly around the body but is concentrated in the trunk [50].

A growing number of studies has demonstrated that cortisol has a key effect on programming after either intra-uterine or childhood dietary restriction [51–53]; it has also been associated with an increase in body fat and, more specifically, with central adiposity [54–56]. An excess of cortisol is associated with profound changes in intermediate metabolism, resulting in long-term changes in lipid metabolism [51,57] and an increase in the concentration of tumoural necrosis factor-alpha (TNF-α) [58]. The number of glucocorticoid receptors is greater in the central adipose tissue compared with the peripheral adipose tissue. Furthermore, cortisol raises the activity of lipoprotein lipase, increasing the rate of plasma fatty acid uptake [59,60].

In addition, data from our group show smaller gains in bone mineral content and bone mineral density during a three-year follow-up period in both undernourished boys and girls (Table 1). These results reveal an important negative impact of undernutrition on bone growth and bone mineral density in children.

## 7. Hypertension

A high prevalence of arterial hypertension has been found in children, adolescents and adults with nutritional stunting. One study [61] that investigated arterial pressure in a random sample of adolescent slum residents with stunting (10–16 y, n = 56) showed an elevated percentage of these individuals to have an arterial pressure above the 90th and 95th percentiles, adjusted for height. Nineteen percent of the boys and 23% of the girls exhibited diastolic hypertension (>95th). Thirty-three percent of the boys and 27% of the girls presented values above those of the 90th percentile and below those of the 95th percentile. In other words, these adolescents were at risk for hypertension. Considering the group of patients as a whole, the prevalence of diastolic arterial hypertension was 21% (95% confidence interval 10%–32%). The prevalence of cases with a systolic or diastolic arterial pressure above the 90th percentile was 51% (95% confidence interval 37%–65%).

Another study [62] done in the northeast of Brazil with 416 adults (18–60 y), also slum residents, showed that arterial hypertension (AH) was prevalent in 28.5% of the population (women = 38.5%; men = 18.4). The systolic and diastolic AH increased according to the reduction in stature, and this disease was more prevalent in women who were obese and short (50%) than in those who were obese but not short (OR = 1.98; CI = 1.22–2.96). Recently, another survey [63] investigated whether the health conditions of mothers who had a short stature were different from those without stunting or that of their offspring. A short maternal stature was independently associated with obesity, abdominal obesity and increased arterial pressure. Furthermore, short maternal stature was associated with a LBW and stunting in children. A series of studies has shown that central adiposity is associated with hypertension and type 2 diabetes [64–66].

**Figure 4.**
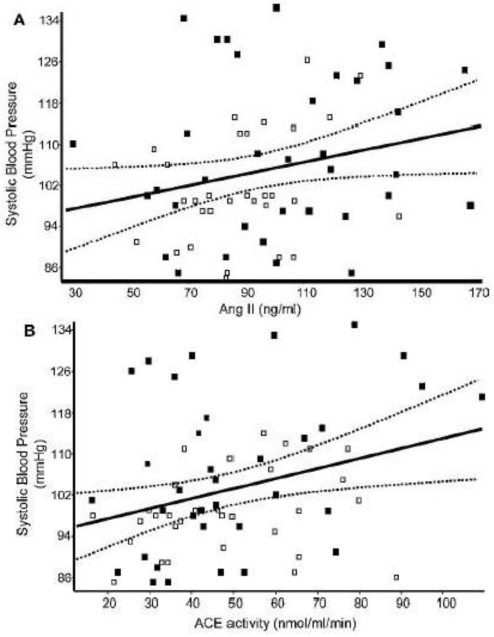
Correlation between SBP and (**A**) AngII and (**B**) ACE activity for girls (open symbols) and boys (solid symbols). The solid lines represent the linear regression and the broken lines are the 95% confidence intervals. Reprinted with permission from *Clin. Sci.* [67].

Franco *et al.* [67] reported changes in the sympathoadrenal and renin-angiotensin systems in children small for their gestational age (SGA). They investigated the plasma levels of ACE (angiotensin-converting enzyme), angiotensin and catecholamines in 8 to 13-year-old children to determine correlations between the plasma levels and both birth weight and blood pressure (BP). Circulating noradrenaline levels were significantly elevated in SGA girls compared to girls born with a weight appropriate for their gestational age. In addition, angiotensin II (AngII) and ACE activity were higher in SGA boys. There was a significant association between the circulating levels of both angiotensin II and ACE activity and systolic BP (SBP, Figure 4). Febba *et al.* [68] recently showed that ACE activity is increased, together with an increase in systolic and diastolic pressure in children with stunting independent of birth weight.

Research in humans and animals suggests that the intra-uterine development of the kidney is particularly affected by maternal undernutrition. This causes a delayed intra-uterine growth and a lower number of nephrons [69]. The renal structure, specifically the number of nephrons, is the main determinant of the blood pressure and renal function in such a way that individuals with a low number of nephrons present arterial hypertension throughout life. There is also evidence of changes in the renal morphology in undernourished individuals, including changes in the size of the organ [70] and a lower number of glomeruli [71].

## 8. Glucose and Insulin Metabolism

The total number of people with diabetes worldwide is projected to rise from 171 million in 2000 to 366 million in 2030, and the ten countries with the highest number of diabetics will be India, China, USA, Indonesia, Pakistan, Brazil, Bangladesh, Japan, the Philippines and Egypt. Of these, only the USA and Japan are developed countries, with the remaining countries all being considered developing countries [72]. Although the prevalence of type 2 diabetes in individuals that were undernourished in early life is not known, it is known that poor countries with an accelerated process of urbanisation are particularly vulnerable and have been experiencing a considerable increase in the prevalence of type 2 diabetes [73]. A study carried out by Fekadu *et al.* [74] in adult diabetic individuals found a significant association between a history of undernutrition and a lack of a clean water supply during childhood, emphasising the importance of adequate post-natal development for the maintenance of health in the long-term.

Deleterious changes have been reported in the metabolism of glucose in children suffering from undernutrition in infancy. One study that examined the effects of undernutrition in the first year of life on glucose tolerance and plasma insulin found that early undernutrition in the extrauterine period, independent of the birth weight, was associated with hyperinsulinemia and a reduced sensitivity to insulin, which worsened as BMI increased in adult life [75].

We examined these hormonal changes in adolescence and observed that the stunted boys and girls showed plasma insulin levels that were significantly lower when associated with a lower homeostasis model assessment-B (HOMA-B), which evaluates pancreatic b-cell function, than those of a nonstunted group [76]. At the same time, their values for HOMA-S (an evaluation of insulin sensitivity) were significantly greater at this age. We suggested that the increase in insulin sensitivity might be due to a higher number of peripheral insulin receptors, especially in the adipose and muscle tissues, which may establish a counter-regulatory mechanism to compensate for the low levels of insulin; such a mechanism could contribute to the preferential increase in fat with age described previously.

Adult women of short stature (height ≥ 25 th percentile) and suffering from obesity were examined to investigate the association between abdominal obesity and resistance to insulin [62]. The results revealed a tendency towards increased insulin resistance, along with impaired glycaemic and lipid profiles, as BMI increased. While an increase in total body mass was associated with a moderate decline in peripheral sensitivity to insulin, abdominal obesity was characterised by a steep decline in such sensitivity and was accompanied by a reduction in glucose peripheral stimulus and a reduction in insulin output. HOMA-IR (insulin resistance) and HOMA-B were significantly higher in the short stature group in comparison to those with average stature. Multivariate logistic regression analysis found that stature was the main factor associated with insulin resistance. In addition, in comparison to women of average stature, short women presented higher levels of glycated haemoglobin, total and low-density lipoprotein (LDL) cholesterol, whereas their high-density lipoprotein (HDL) cholesterol levels were significantly lower.

The literature contains a growing number of studies that have linked pancreatic function to undernutrition in early life [75,77,78]. Apparently, the lower beta cell function is due to a lower beta cell number as a result of malnutrition. Furthermore, this could be a consequence of the increased concentration of glucocorticoids that occurs in undernutrition, as normal levels of glucocorticoids are necessary to ensure the development and maintenance of normal pancreatic architecture, as well as the expansion of the beta cell mass during critical periods of development [78]. Based on the results described above it, appears plausible that in adolescence, a decrease in the function of the pancreatic cells is compensated by an increase in sensitivity to insulin. However, as the amount of abdominal fat and age increase, this condition begins to place more intense demands on undernourished individuals, causing an increase in pancreatic activity, which accelerates the exhaustion of the organ and the onset of diabetes [73,76].

## 9. Undernutrition, Stress and Impaired Mental and Living Conditions

Undernutrition is the most powerful condition of physiologic stress, because it is life threatening [79]. Stress invokes two main mechanisms of response in the organism: the axis formed by the sympathetic nervous system, the adrenal medulla (SAM), which stimulates the synthesis and release of adrenaline and noradrenaline, and the HPA axis, which acts by stimulating the production of cortisol. In addition to the fight-or-flight reaction to acute stress, various events in daily life, such as the nutritional stress that results from disturbances in the provision of food of adequate quantity and quality, can induce chronic physiologic stress, and over time, this can lead to weakening of the body and even death.

Numerous investigations have shown marked changes in the function of the autonomic nervous system in undernutrition. For example, a study that compared normal and undernourished children in India from 5 to 10 years of age [80], employed tests to assess the function of the parasympathetic nervous system (resting heart rate and the ratio between the measurements obtained lying down and standing, as well as during expiratory pressure for 15 seconds—known as the Valsalva ratio), and the function of the sympathetic nervous system (measurement of blood pressure during static exercise by means of the execution of a test in which the individual exerted pressure with their hand on a determined object for one minute, and a test to measure the galvanic skin response). The results showed that the resting heart rate was significantly higher in the undernourished group; likewise, the tests to examine the parasympathetic function suggested that this was compromised in these children. The exams designed to assess the sympathetic activity demonstrated a change in systolic and diastolic arterial pressure as well as in the galvanic skin response in the undernourished children, indicating an increase in sympathetic tone.

Classical studies on marasmic children with kwashiorkor showed that increased blood cortisol concentrations were positively associated with the severity of the disease and the degree of infection [81,82]. In village studies in Uganda, plasma cortisol [83] rose as those of insulin and albumin fell, indicating that nutritional status alone can modulate cortisol production. Another classical study, however, that measured plasma cortisol in undernourished children showed that cortisol concentration did not differ from those of the well-nourished children, either before or after 15 or 30 days of treatment, unless there was a superimposed acute stress [84]. One plausible explanation for these differences in plasma cortisol is presented in a publication by Fernald *et al.* [85]. Based also on animal studies, the authors discussed that different stressful conditions cause either blunted cortisol or “hypocortisolism” and heart rate responses or, on the contrary, increased cortisol and heart rate. It seems to depend on the type, duration and severity of the stress. Long-lasting exposure to stress seems to lead to a blunted salivary cortisol response and acute stress-increased HPA reactivity.

It is well known that cortisol promotes muscle proteolysis and hepatic protein synthesis, and these metabolic effects enhance the acute phase response, an essential component of the successful host response to infection. However, these protein metabolic and acute-phase responses are blunted in marasmic children [82]. Cortisol exerts its acute effect via the glucocorticoid receptor present in the cytosol. The receptor, upon binding with this hormone, dissociates from other proteins, undergoes phosphorylation and translocates to the nucleus. The cortisol/receptor complex can then bind to specific DNA sequences and, in conjunction with coactivators, promote transcription of some genes and repress the expression of others. These actions are diminished after a few days by glucocorticoid resistance, which is achieved by the down-regulation in the number of glucocorticoid receptors, the expression of an inactive isoform of the receptor and repression of phosphorylation of the hormone/receptor complex by a transcription factor nuclear factor [86]. A study that investigated marasmic children found no reduction in the whole-cell glucocorticoid receptors in the sick children with or without infection, but higher nuclear glucocorticoid receptors in leucocytes compared to well-nourished children with infection [82]. The authors concluded that an increased expression of 11β-hydroxysteroid dehydrogenase type 1 (an enzyme capable of increasing intracellular cortisol independently of circulating concentrations) as a consequence of marasmus could result in the increased nuclear glucocorticoid receptors. Unfortunately, these authors did not measure plasma cortisol in control well-nourished children without infection for proper comparison of hormone concentrations. Recent studies in experimental animals found increased glucocorticoid hypothalamic receptor expression in the fetus associated with maternal undernutrition, indicating an epigenetic effect of this hormone [87]. Evidence of modifications of the epigenetic status of the glucocorticoid receptor was found in the liver from offspring of rats that were fed a protein-restricted diet throughout pregnancy [88]. When compared to the control offspring, the diet-restricted offspring had decreased glucocorticoid receptor methylation, with a 200% increase in glucocorticoid receptor expression. The increase in glucocorticoid receptor expression suggests that there is an increased physiological influence of this hormone. Furthermore, the changes in glucocorticoid receptor methylation persisted in the offspring even though the dietary restriction had stopped, suggesting that the methylation status of genes is potentially permanent [88,89]. This study indicates that maternal nutritional insults can alter the methylation patterns of glucocorticoid receptor in the fetus. Cortisol release is the first metabolic response to psychological stress and the hypersecretion of this hormone is observed in depression, in work stress and among men of low income [90]. In addition, precarious living conditions, such as poverty, which increase the prevalence of depression and anxiety, are also responsible for a greater release of cortisol. A relationship has been demonstrated between socio-economic factors and an increased risk of coronary disease and type II diabetes [74,90]. These psychosocial factors stimulate the activity of the mechanisms of the stress response and are at least partly responsible for the increased frequency of these diseases. On the other hand, studies have shown that people who describe themselves as happy have lower levels of cortisol, as well as lower heart rates and lower levels of fibrinogen, in response to stressful stimuli [91].

The evolution of studies on the effects of stress on the organism has provided evidence that stress at the beginning of life, whether it be physiological or psychological, or as is the case for the great majority of people, the combination of these two factors, results in an epigenetic effect, altering neurogenesis, cerebral plasticity and the brain's capacity for regeneration, the formation of dendritic trees and the number of synapses, in addition to affecting cognitive function, appetite control, and the amount of body fat, among many other parameters [92]. Neglect at the beginning of life and precarious or insufficient care is associated with permanent changes in the mechanisms of stress control, raising vulnerability to stress in adult life and causing hypersensitivity to stressful episodes, which alters the fear response, reduces the cognitive capacity, increases vulnerability to the consumption of alcohol, *etc.* Maternal behaviour can effectively change the genetic control of stress. The genetic information linked to the neuroendocrine response to stress can be programmed by maternal stimulation. In experimental models, intense maternal behaviour promotes a neurobiology of stress that is less reactive and more resilient to challenges in the future life of the offspring. The mechanisms are highly specific and involve relatively permanent modifications in the control of the expression of genes encoding glucocorticoid receptors. Studies have shown that stress-induced modifications can be reversed in the presence of positive and socially stimulating environments [93]. The presence of caregivers who are affectively linked to the children can prevent a rise in cortisol (in breast-feeding mothers and children), even in the presence of external threats. This response allows children to ask for help and express negative emotions without activating this endocrine component of the response to stress. Inversely, when paternal behaviour is insufficient and/or is the source of the threat, relationships can be an important source of stress detected physiologically through the increase in cortisol and other factors in children and animals [93].

One study examined the long-term effects of perinatal undernutrition (a reduction of 50% in food intake), from the 14th day of pregnancy until the end of lactation, on the activity of the hypothalamus– pituitary–adrenal axis and the sympathoadrenal catecholaminergic system in basal conditions and in conditions of stress caused by restraint for two hours in adult rats and found that undernutrition lead to a much more marked impact of corticosterone on the target cells responsive to stress in relation to the control rats [94]. Another investigation of the same kind of perinatal undernutrition showed atrophy of the adrenal gland with a negative impact on the activity of the HPA axis and the differentiation of adrenomedullary cells, as well as on the nerves. These changes were associated with adaptive reprogramming of the metabolic response to stress [95]. Men and women exposed to starvation during gestation as a consequence of the Dutch famine that occurred during the Second World War presented an increased activation of the HPA axis in response to psychological stress; however, no differences were found in the basal and peak concentrations of cortisol when the study participants were compared with others examined before and after the famine [96].

Stunting has been linked to poor mental development and school achievement, and behavioural abnormalities in children [97]. However, there is still a debate in the literature on whether these effects are permanent or can be reversed. In national data using Dutch military recruits examined at an age of 18 y [98] no association was found between prenatal famine exposure and the prevalence of either mild or severe mental retardation or a decrease in IQ, as estimated from the mean score on the Raven progressive matrices test. The authors argued that because of the nature of the sample and the number of individuals available for the study, the findings make a convincing case for the absence of long-lasting effects up to young adulthood, at least in males. In a further examination of cognitive function among 971 men and women aged 59 years in one of the Dutch famine birth cohorts, individuals exposed to famine during gestation did not differ in cognitive outcomes compared with the controls born before or conceived after the famine or compared with unexposed same-sex siblings [99]. On the other hand, in a separate but comparable Dutch famine birth cohort including 737 men and women examined at the same age, an association was reported between prenatal famine and a selective attention task [100]. In the Dutch studies, prenatal famine exposure has also been related to an increase in schizophrenia risk and other psychiatric disorders in adulthood [100].

A recent study using longitudinal data on 1,674 Peruvian children measured the effects of recovery from early stunting on levels of cognition [101]. The authors evaluated children that were 6–18 mo. of age and again when they were 4.5–6 y of age. They found that children who had grandparents in the home and had taller mothers had less severe stunting in infancy and were more likely to demonstrate linear catch-up by around 2. Children who experienced catch-up growth had a verbal vocabulary and quantitative test scores that did not differ from children who were not stunted. The authors concluded that children can recover from an early nutritional insult and improve in cognition performance.

Undernutrition also has consequences for the individual's capacity for physical labour. A low BMI is related to a greater number of absences from work and also to lower productivity [102,103]. A BMI of 17 kg/m^2^ appears to be critical for the capacity for work, and below this value, productivity is negatively impacted. Light activities, such as planting crops, hoeing and cutting grass do not appear to be negatively affected when performed by people with a BMI below 17 kg/m^2^. On the other hand, heavy activities such as cutting sugar cane, loading or carrying sacks of grain or cotton, digging earth or coal, using a sledge-hammer to hammer in fence posts, pulling or cycling a rickshaw, stone splitting and pushing a loaded wheel-barrow, which not only involve high energy expenditure but also require the use of the body mass, are markedly affected by undernutrition [103]. Our group investigated the productivity of sugar cane harvesters according to their nutritional status [104] and observed that workers with a BMI within the normal range were more productive and consumed a significantly greater amount of energy compared to those employees with a BMI below 21.5 kg/m^2^ or above 25 kg/m^2^ (Table 2).

Taller workers presented higher productivities and tended to consume more carbohydrates and proteins while those who were shorter tended to have more body fat and a lower productivity (Table 3). In a multiple regression analysis, stature was identified as the parameter most closely associated with productivity, after adjusting for age and the proportion of body fat.

**Figure 5.**
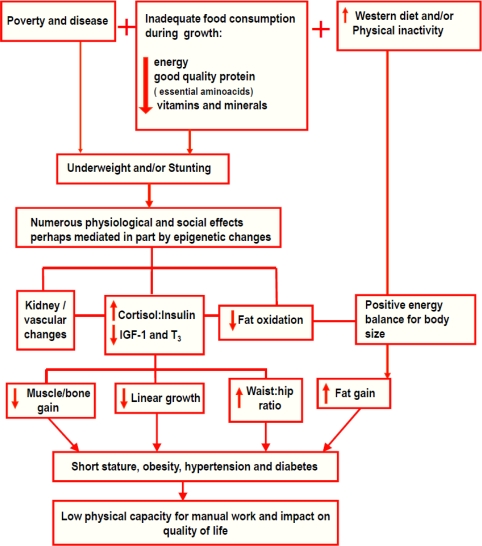
Association between short stature, obesity, hypertension, diabetes and work capacity.

In essence, undernutrition, if it does not lead to death, has very negative consequences on individual health and standard of living individual throughout the life cycle. The same is true for a country's economy. Undernutrition is caused by poverty and disease, mainly diarrhoea, respiratory infections and parasites, associated with inadequate food consumption during growth (insufficient energy, good quality protein with balanced essential amino acids, vitamins and minerals) (Figure 5). As described previously, undernutrition (underweightness and/or stunting) leads to numerous physiological and social alterations, perhaps mediated, in part, by epigenetic changes. It is a powerful stimulator of stress and can prompt an increased secretion of cortisol leading to an increase in the cortisol-to-insulin ratio to direct energy in the form of glucose to the brain. This hormonal balance leads to a reduction in key hormones responsible for growth, such as IGF-1, and thyroid hormones, leading to lower linear growth and lower energy expenditure. In addition, energy restriction reduces anabolic events in insulin-dependent tissue synthesis, resulting in lower lean body mass and impaired bone growth. On the other hand, the redirecting of body energy flow favours the accumulation of abdominal fat, increasing the waist-to-hip ratio, as well as causing a reduction in body fat oxidation. Undernutrition negatively affects the formation of various tissues and organs, with the pancreas, kidneys and the vascular systems appearing to be particularly affected. If a child in such a condition begins to ingest a ‘modern western’ diet rich in industrialised food and presents physical inactivity due to urban living conditions, a positive energy balance for their body size and consequently, an excessive fat gain will take place, which may result in the association between stunting, obesity, hypertension and diabetes, as well as impaired working capacity, causing an impact on the quality of life, as shown in this review.

## 10. Nutritional Recovery

One of the biological variables with the greatest impact on the long-term health of undernourished children is the recovery of stature. For this reason, special attention to the quality of the diet during nutritional recovery is fundamental, especially the quality of protein and the essential amino acids consumed, to enable a gain in stature without an unwanted increase in energy provision that might favour the later development of obesity. As an example, a study in children of school age [105] provided a protein-rich diet to one group while a second group received a diet with added oil. The group given a protein-rich diet exhibited an increase in height directly related to the quantity of supplementary protein, while no detectable effect was present in the group consuming a diet with added oil. It was also shown that the quantities of both protein and energy are important in the regulation of IGF-1, because these factors were essential in the restoration of the serum levels of IGF-1 [34,36]. In this study, refeeding with a normocaloric and normoproteic diet after 5 days of fasting raised the levels of IGF-1 by up to 70% above basal levels before food restriction; meanwhile, refeeding with an isocaloric but hypoproteic diet delayed the recovery in the levels of IGF-1 by 2 days, and the levels of this hormone failed to reach 50% of the values before restriction. In addition, refeeding with a low calorie and low protein diet for more than 5 days reduced the levels of IGF-1 even further.

One strategy to combat childhood undernutrition is investing in the creation of centres of recovery for undernourished children (0–6 y) with outpatient care as well as a day-hospital. Our groups at Federal University of São Paulo and Federal University of Alagoas established a series of Centre for Nutritional Recovery and Education (CREN) units to develop nutritional rehabilitation methodologies in both scientific and social contexts to guarantee long-lasting positive impacts on nutritional recovery in poor urban areas. A description of the health and nutritional profile of the children treated at the CREN in São Paulo was published recently [106]. Among the 106 children studied, 92.5% recovered at least one anthropometric index and 67.9% recovered in both weight and height. Almost half of the children presented a nutritional recovery of more than 0.50 in HAZ (46.2%) and about 40% in weight-for-age (WAZ) (38.7%). The mean increment in WAZ, HAZ and weight-for-height (WHZ) of all children studied from admission to discharge was 0.476, 0.508 and 0.193, respectively. The average age of the children at admission was 23.7 months with an equal proportion of boys and girls. Neither age nor gender was associated with the nutritional recovery pattern. One of the most important findings described was that catch-up in stature occurred in children older than the age of 2 y, and no differences were found among groups with <2 y and >2 y in terms of height recovery. The authors discussed that age becomes less important in terms of nutritional recovery if treatment is done adequately, although this finding demands more investigation to be validated. The average duration of treatment was 16.4 months for all children and a longer treatment was associated with a greater gain in both WAZ and HAZ. The mean birth weight of all children was 2,563 g and approximately 40% of the children were classified as LBW. The height gain was significantly different according to birth weight, being greater among those who were born smaller; the same was not found for weight gain. The frequency of SGA was significantly greater among children who presented greater height gains. Considering nutritional status at admission, only WAZ was significantly associated with weight gain. However, height gain was associated with both WAZ and HAZ at admission. The most prevalent diseases during treatment were upper respiratory tract infection (URTI), which 82% of the children developed at least once, while 44% had diarrhoea and 18% had lower respiratory tract infection (Table 4).

A study was performed with the objective of determining the body composition of previously malnourished children who adequately recovered in both height and weight after being treated at the CREN [107]. Children and adolescents, aged 4–14 y, were divided into 3 groups. The control group consisted of subjects who had, at the time of the study, weight-for-age Z-scores ranging from 0 to +1.645 and height-for-age Z-scores from 0 to +2, living in the same neighbourhood as the other groups. The outpatient group consisted of recovered subjects, with weight-for-age and height-for-age Z-scores ranging from −2 to −1.645 when admitted for treatment at the CREN.

The day-hospital group consisted of recovered children, who had weight-for-age Z-scores and height-for-age Z-scores lower than −2 when admitted for treatment in the day-hospital at CREN. At follow-up, both treated groups had similar weight-for-age Z-scores and height-for-age Z-scores, but these scores were lower than those of the controls. Nevertheless, the recovered groups were above the cut-off value for normal height (74% and 79% of the outpatient and day-hospital group had Z-scores above −1.00, respectively) and weight (56% and 58% of the outpatient and day-hospital group had Z-scores above −1.00, respectively). Among prepubertal children, body fat mass (kg) and body fat percentage were significantly lower in both recovered groups of girls and boys compared with the controls. The day-hospital boys also had a significantly lower percentage of body fat than the outpatient boys. Lean mass per unit of height (kg/cm) and the fat-free mass index (kg/m^2^) were significantly lower in the recovered boys compared with the controls, but did not differ in girls. When all of the children were analysed (including the pubertal children), all of the parameters assessed for body composition were lower in the recovered groups compared with the controls for both boys and girls, except for percentage of body fat, because the boys in both recovered groups had percentages similar to those of the boys in the control group. Bone mineral content/height (BMC, g/cm) did not differ between the recovered girls and girls in the control group or between the boys in the day-hospital group and those in the control group. The outpatient boys group had a lower BMC/height than the boys in the control group. The protein intake was adequate in the 3 groups, but the recovered groups consumed significantly more protein than the control group. This study demonstrates that when malnourished children receive adequate treatment, linear catch-up occurs and is followed by appropriate gain in body mass and BMC.

Another study [108] with children who recovered from undernutrition was performed to determine HOMA-S and HOMA-B from fasting plasma glucose and insulin concentrations. The mean concentration of plasma insulin adjusted for age and pubertal stage did not differ between the control and the recovered groups for boys and girls. There was also no difference in glucose concentration between the groups. The control and the recovered groups did not present differences in HOMA-B. The results were also similar for the HOMA-S index (Table 5). This may be explained by the fact that the foetal and post-natal periods are fundamental for the development of pancreatic beta cells as well as the maturation of their function. Therefore, if there is adequate recovery from undernutrition before the age of 6 y, the changes in pancreatic metabolism described in undernutrition appear to be reversed.

## 11. Conclusions

Undernutrition is still a major problem in the World. Of particular concern is the described coexistence of undernutrition and obesity in many developing countries. The rapid transition in these countries has manifested this double burden of disease, which is clearly seen in a family with an undernourished child of obese parents, representing two phases of the dual nutritional insult within the same environment. This situation aggravates the costs for the public health system and the poverty of the country, impairing its ability to overcome poverty.

The current review shows that some progress has been made over the past decades to highlight the long-lasting effects of early undernutrition. The most consistent findings are an increased susceptibility to accumulate fat mostly in the central region of the body, lower fat oxidation, lower energy expenditure, insulin resistance and a higher risk of diabetes in adulthood, hypertension, dyslipidaemia and a lowered working capacity of manual workers, among other physiological impairments. Studies in this area are very difficult to perform because they should consider the whole life-course of the individuals and can only be done in experimental models because of ethical reasons.

A much less clear picture is related to the effects of undernutrition and mental health. On one hand, difficulties in achieving reliable and valid measurements of mental development and behaviour in poor children and allowing for the confounding and possibly interacting effects of social background have largely been addressed in the literature. On the other hand, there are very relevant studies showing that children in Peru who recovered from early stunting demonstrated normal levels of cognition in comparison to nonstunted counterparts. Studies in this area and on the risk of the epigenetic effects of undernutrition are urgent and very important.

There is still much debate over whether programming or epigenetic modifications persist over multiple generations. If maternal undernutrition can exert major effects on fetal development and future disease pattern, this knowledge is of great importance to public health. For this reason, the results presented in children recovered from undernutrition before 6 y of age in day-hospitals and outpatient clinics are particularly relevant. The results presented here showed a recovery in weight and height, normal body composition, bone mineral density and insulin production and sensitivity. Further work remains necessary to investigate other positive effects of post-natal nutritional recovery.

Finally, programs and policies should be designed to prevent undernutrition as well as its long-lasting effects and should be addressed particularly to maternal health and education, and these programs should include a diet of good quality for pregnant women. Additionally, this review presents evidence of post-natal nutritional recovery that include older children (>2 y).

## Figures and Tables

**Table 1.** Gains in bone mineral content and bone mineral density, according to nutritional status and sex.

	
	**Boys**		**Girls**	
	**Eutrophic** (n = 13)	**Undernourished** (n = 12)	**Eutrophic** (n = 17)	**Undernourished** (n = 8)
**Bone mineral content**				
Arms (g)	46.44 ± 16.90	21.63 ± 9.99 **	48.77 ± 21.31	30.74 ± 13.40 *
Legs (g)	154.03 ± 45.03	35.65 ± 165.06 *	127.17 ± 42.79	73.83 ± 18.05 **
Trunk (g)	135.34 ± 103.64	67.62 ± 22.79 *	169.14 ± 66.95	82.43 ± 75.06 *
Total (g)	557.86 ± 206.66	275.34 ± 107.69 **	570.87 ± 211.26	330.85 ± 133.78 *
**Bone mineral density**				
Arms (g/cm^2^)	0.054 ± 0.047	0.027 ± 0.026	0.067 ± 0.042	0.037 ± 0.042
Legs (g/cm^2^)	0.209 ± 0.080	0.108 ± 0.057 *	0.184 ± 0.062	0.094 ± 0.030 **
Total (g/cm^2^)	0.099 ± 0.059	0.038 ± 0.043 *	0.114 ± 0.058	0.051 ± 0.048*

Values expressed are mean ± standard deviation;

* Significantly different from the eutrophic group, p < 0.05;

** Significantly different from the eutrophic group, p < 0.001.

**Table 2.** Productivity, energy intake and nutrients according to BMI of sugarcane cutters studied at a plantation in Marechal Deodoro (Alagoas, Brazil).

**BMI (kg/m^2^)**	**Number of individuals n (%)**	**Average (±SD) productivity (ton/day)**	**Average (±SD) energy intake (kJ/day)**	**Average (±SD) daily nutrient intake (g/kg body weight)**		
				**Proteins**	**Carbohydrates**	**Lipids**

<21.5	25(40.3%)	7.48 ± 1.5	12,380 ± 4,184	2.0 ± 0.5	7.4 ± 3.6	0.8 ± 0.6
21.5–25	30 (48.4%)	9.12 ± 1.5 *	16,506 ± 6,360 *	2.1 ± 1.5	9.8 ± 4.1 *	1.7 ± 0.7 *
>25	7 (11.3%)	7.80 ± 1.7	13,215 ± 1,251	2.0 ± 0.5	8.5 ± 1.4	1.2 ± 0.3

* ñ < 0.05; reprinted with permission from *Arch. Latinoamer. Nutr.* [104].

**Table 3.** Productivity, intake of energy and proteins, body fat composition and average age of sugarcane cutters at a plantation in Marechal Deodoro (Alagoas, Brazil) distributed according to height groups.

**Height (cm)**	**Number of individuals n (%)**	**Average age (years)**	**Average productivity (ton/day)**	**Average daily energy intake (kJ/day)**	**Average daily protein intake (g/kg body weight)**	**Body fat composition**		
						**Initial (a) (%)**	**Final (b) (%)**	**Loss Δ = a−b**

158–159.9	12 (19.4%)	42 ± 12.3	7.14 ± 1.6 *	14,292 ± 4,300	2.2 ± 0.7	17.7 ± 4.3	16.6 ± 5.7	1.1
160–164.9	16 (25.8%)	35 ± 10.7	7.90 ± 1.7	11,593 ± 4,449	1.8 ± 0.7	13.7 ± 2.2	12.9 ± 2.0	0.8
165–169.9	12 (19.4%)	37 ± 10.3	7.95 ± 1.5	12,041 ± 3,969	1.9 ± 0.7	13.3 ± 3.6	12.4 ± 4.3	0.9
170–174.9	12 (19.4%)	30 ± 8.6	9.01 ± 1.5 *	15,388 ± 5,217	2.4 ± 0.5	12.3 ± 3.5	11.4 ± 2.5	0.9
=175	10 (16.1%)	26 ± 7.4	8.65 ± 1.4	15,551 ± 6,758	2.2 ± 0.7	9.8 ± 2.5	9.5 ± 1.9	0.3

* p < 0.05; reprinted with permission from *Arch. Latinoamer. Nutr.* [104].

**Table 4.** Characteristics of weight and height gain according to the studied variables among children (n = 106) aged 0 to 72 months living in southern Sao Paulo, Brazil, who attended a daily care service.

	
	**Increment of WAZ at discharge**				***P***	**Increment of HAZ at discharge**				***P***
	**≤0 (*n*21)**	**0.01–0.50 (*n*44)**	**0.51–1.00 (*n*22)**	**>1.00 (*n*19)**		**≤0 (*n*21)**	**0.01–0.50 (*n*36)**	**0.51–1.00 (*n*28)**	**>1.00 (*n*21)**	
Age at admission (months), median *	17.4	17.5	20.9	24.2	0.527	10.8	21.4	23.2	19.1	0.338
Gender (% male) †	57.1	47.7	59.1	36.8	0.464	66.7	50.0	42.9	42.9	0.343
Duration of treatment (months), mean ‡	10.1	14.8	19.7	23.0	<0.001	9.2	12.9	22.2	21.7	0.957
Fetal development (% premature) †	20.0	18.4	25.0	29.4	0.811	21.1	25.9	20.0	21.1	0.957
Birth weight (kg), mean ‡	2.593	2.648	2.550	2.317	0.103	2.670	2.619	2.604	2.295	0.045
Small for gestational age (%) §	27.8	21.4	19.1	50.0	0.129	21.1	18.2	23.1	52.6	0.026
WAZ at admission, mean ‡	−2.04	−2.08	−2.49	−2.52	<0.001	−2.22	−2.07	−2.26	−2.51	0.041
HAZ at admission, mean ‡	−1.92	−1.91	−2.36	−2.26	0.061	−1.63	−1.78	−2.27	−2.71	<0.001
Frequent URTI (rate 10–3) *	55.3	26.2	44.1	33.4	0.011	22.9	36.8	37.3	39.3	0.226
Frequent LRTI (rate 10–3) *	5.8	2.4	1.3	0.5	0.857	1.8	3.7	1.3	1.7	0.712
Frequent diarrhoea (rate 10–3) §*	11.2	5.6	6.0	6.0	0.215	7.1	10.7	3.5	6.0	0.201
Maternal education (years), mean ‡	2.1	2.4	2.3	2.3	0.767	2.5	2.3	2.1	2.3	0.347
Family income (R$), median*	300	300	352	268	0.454	350	300	300	300	0.721

WAZ, weight-for-age Z-score; HAZ, height-for-age Z-score; URTI, upper respiratory tract infection; LRTI, lower respiratory tract infection;

* Kruskal–Wallis test;

† x^2^ test;

‡ One-way ANOVA test for linear trend;

§ x^2^ test for linear trend; Reprinted with permission from *Public Health Nutr.* [106].

**Table 5.** Insulin, homeostasis model assessment of pancreatic b-cell function (HOMA-B), homeostasis model assessment of insulin sensitivity (HOMA-S) and glucose concentration for boys and girls in the control and recovered groups (Mean values with their standard errors).

	
	**Boys**						***p*†**	**Girls**						***p***
	**Control**			**Recovered**				**Control**			**Recovered**			

	**n**	**Mean**	**SE**	**n**	**Mean**	**SE**		**n**	**Mean**	**SE**	**n**	**Mean**	**SE**	
Insulin (pmol/L) *	15	3.62	0.40	28	3.76	0.28	NS	9	3.78	0.26	30	3.52	0.09	NS
Glucose (mg/dL)	15	78,90	5.92	27	73.28	4.23	NS	9	75.54	4.38	29	79.04	1.65	NS
HOMA-B (%)*	15	4.71	0.308	27	4.92	0.220	NS	9	4.84	0.206	29	4.59	0.076	NS
HOMA-S (%)*	15	4.85	0.403	27	4.75	0.288	NS	10	4.73	0.264	30	4.96	0.098	NS

NS, P > 0.05;

* Logarithmically transformed;

† From analysis of covariance with means adjusted for age and pubertal stage; Reprinted with permission from *Brit. J. Nutr.* [108].

## Abbreviations

**Table:** Table of Acronyms (in alphabetical order)

ACE	Angiotensin-Converting Enzyme
ACTH	Adrenocorticotropic Hormone
AH	Arterial Hypertension
AngII	Angiotensin II
BMC	Bone Mineral Content
BMD	Bone Mineral Density
BMI	Body Mass Index
BP	Blood Pressure
CREN	Center For Nutritional Recovery And Education
CRH	Corticotrophin Releasing Hormone
DALYs	Disability-Adjusted Life Years
DXA	Dual-Energy X-Ray Absorptiometry
GH	Growth Hormone
HAZ	Height-for-Age
HDL	High-Density Lipoprotein
HOMA	Homeostasis Model Assessment
HPA	Hypothalamic Pituitary Adrenocortical System
IGF-1	Insulin-Like Growth Factor type 1
LBM	Lean Body Mass
LBW	Low Birth Weight
LDL	Low-Density Lipoprotein
LRTI	Lower Respiratory Tract Infection
OR	Odds Ratio
RDA	Recommended Dietary Allowances
RMR	Resting Metabolic Rates
RQs	Respiratory Quotient
SAM	Sympathetic Adrenomedullary (System)
SBP	Systolic Blood Pressure
SGA	Small for Gestational Age
TEE	Total Energy Expenditure
TNF- α	Tumoral Necrosis Factor - Alpha
TRH	Thyrotropin Releasing Hormone
TSH	Thyroid Stimulating Hormone
URTI	Upper Respiratory Tract Infection
WAZ	Weight-for-Age
WHZ	Weight-for-Height
